# Medical Society of Virginia

**Published:** 1882-09-20

**Authors:** 


### *MEDICAL SOCIETY OF VIRGINIA.*

The transactions of the Medical Society of the State of Virginia should have been earlier noticed. The last meeting was held on October 10th, 11th and 12th, 1881. The proceedings were published in the January number of the Virginia Medical Monthly, and makes 173 pages of that issue.

The President, Dr. Hunter McGuire, very pleasantly departs from the usual routine, and makes the Annual Address upon a special subject—Clinical Remarks on Cancer of the Breast. The address is an able one.

The papers read before the body nearly all evince ability and research. They were as follows:

Report on advances in Anatomy, by Christopher Tompkins, M. D.

Report on the Advances in Surgery, by Meade C. Kemper, M. D.

REPORT ON ADVANCES IN PRACTICE OF MEDICINE, by Bedford Brown, M. D. Catarrhal Deafness, by J. A. White, M. D. Emetic effect of Chloroform administered by deglutition, and the advantages of Hydro Bromate of quinine hypodermically, by G. W. Semple, M. D. Physiological and therapeutic action of the sulphate of quinine, by Otis F. Manson, M. D.

President elect, G. W. Semple, of Hampton. Recording Secretary, L. B. Edwards, Richmond. Place of next meeting, Fauquir White Sulphur Springs, Va.

Dr. S. B. Morrison, of Brownsburg, will deliver the Annual Address to the Public.

